# Towards systems genetic analyses in barley: Integration of phenotypic, expression and genotype data into GeneNetwork

**DOI:** 10.1186/1471-2156-9-73

**Published:** 2008-11-18

**Authors:** Arnis Druka, Ilze Druka, Arthur G Centeno, Hongqiang Li, Zhaohui Sun, William TB Thomas, Nicola Bonar, Brian J Steffenson, Steven E Ullrich, Andris Kleinhofs, Roger P Wise, Timothy J Close, Elena Potokina, Zewei Luo, Carola Wagner, Günther F Schweizer, David F Marshall, Michael J Kearsey, Robert W Williams, Robbie Waugh

**Affiliations:** 1 Genetics Programme, Scottish Crop Research Institute, Invergowrie, Dundee DD2 5DA, UK; 2 School of Computing and Creative Technologies, University of Abertay, Dundee, DD1 1HG, UK; 3 Department of Anatomy and Neurobiology, University of Tennessee, Memphis TN 38163, USA; 4 Department of Plant Pathology, University of Minnesota, St. Paul, MN 55108, USA; 5 Department of Crop and Soil Sciences, Washington State University, Pullman WA99164, USA; 6 Corn Insects and Crop Genetics Research, USDA-ARS, Iowa State University, Ames IA 50011, USA; 7 Department of Plant Pathology, Iowa State University, Ames IA 50011, USA; 8 Department of Botany and Plant Sciences, University of California, Riverside, CA 92521, USA; 9 School of Biosciences, University of Birmingham, Birmingham B15 2TT, UK; 10 Department of Plant Breeding, Justus Liebig University Giessen, Heinrich-Buff-Ring 26-32, 35392 Giessen, Germany; 11 Institute for Crop Production and Plant Breeding, Dep. Genome Analysis, Bavarian State Research Center for Agriculture, Am Gereuth 6, 85354 Freising-Weihenstephan, Germany

## Abstract

**Background:**

A typical genetical genomics experiment results in four separate data sets; genotype, gene expression, higher-order phenotypic data and metadata that describe the protocols, processing and the array platform. Used in concert, these data sets provide the opportunity to perform genetic analysis at a systems level. Their predictive power is largely determined by the gene expression dataset where tens of millions of data points can be generated using currently available mRNA profiling technologies. Such large, multidimensional data sets often have value beyond that extracted during their initial analysis and interpretation, particularly if conducted on widely distributed reference genetic materials. Besides quality and scale, access to the data is of primary importance as accessibility potentially allows the extraction of considerable added value from the same primary dataset by the wider research community. Although the number of genetical genomics experiments in different plant species is rapidly increasing, none to date has been presented in a form that allows quick and efficient on-line testing for possible associations between genes, loci and traits of interest by an entire research community.

**Description:**

Using a reference population of 150 recombinant doubled haploid barley lines we generated novel phenotypic, mRNA abundance and SNP-based genotyping data sets, added them to a considerable volume of legacy trait data and entered them into the GeneNetwork . GeneNetwork is a unified on-line analytical environment that enables the user to test genetic hypotheses about how component traits, such as mRNA abundance, may interact to condition more complex biological phenotypes (higher-order traits). Here we describe these barley data sets and demonstrate some of the functionalities GeneNetwork provides as an easily accessible and integrated analytical environment for exploring them.

**Conclusion:**

By integrating barley genotypic, phenotypic and mRNA abundance data sets directly within GeneNetwork's analytical environment we provide simple web access to the data for the research community. In this environment, a combination of correlation analysis and linkage mapping provides the potential to identify and substantiate gene targets for saturation mapping and positional cloning. By integrating datasets from an unsequenced crop plant (barley) in a database that has been designed for an animal model species (mouse) with a well established genome sequence, we prove the importance of the concept and practice of modular development and interoperability of software engineering for biological data sets.

## Background

The systems genetics approach coined 'genetical genomics' aims to decompose phenotypic variation into a series of individual components by simultaneously analysing both 'trait' and 'molecular phenotype' data across genetically defined populations. The approach was originally tested by Damerval et al. in 1994 who applied protein profiling to an F2 population of maize [1]. More recently, genetical genomics has been applied to a range of species using microarray derived mRNA abundance phenotypes [2,3]. In mouse, such analyses have been used to understand how regulatory networks controlling transcription relate to higher-order phenotypic traits at the genome-wide scale [4,5]. Analogous genetical genomics experiments in plants have been reported for maize [3,6], Arabidopsis [7,8], eucalyptus [9,10], poplar [11], wheat [12] and barley [13]. These experiments demonstrate that the control of gene expression is complex. However, they also can provide insight into the relationships between gene expression and phenotypic traits.

Genetical genomics experiments typically incorporate four separate data sets for each individual in a segregating population; genotype, mRNA abundance, phenotype and associated metadata. When the genetic materials are 'reference strains' that have been analysed by a broad community, there is an opportunity to incorporate legacy phenotypic and genotypic information. While the scale of the mRNA abundance datasets largely determine the predictive power of the approach, a key point is that these large, multidimensional datasets have considerable value beyond that extracted during their initial analysis. This was recognized early by the scientific community and is formally reflected in regulations specifying raw data quality and availability (archiving) by many funding agencies and journals [14]. However, easy access to the data, either raw or processed, is an equally important criterion that may significantly extend its potential usefulness and value [15,16]. The sheer volume of the genetical genomics data components, if deposited in an open access but unprocessed and in a format designed for archiving, is likely to be of limited value, particularly if only a subset of the data is required for a specific analytical query.

We conducted a genetical genomics experiment in barley using a population of 150 doubled haploid lines [17]. The outcomes of this experiment included two mRNA profiling data sets, a Transcript Derived Marker (TDM)-based barley genetic linkage map and a set of new trait data obtained from over 4 years of field and glasshouse experiments. We also compiled publicly available trait segregation data that has been collected on this reference population by the barley genetics community over the last 15 years. Here we provide open access and availability to these data by integrating them into the GeneNetwork, a web-based analytical tool that has been designed for multiscale integration of networks of genes, transcripts and traits and optimized for on-line analysis of traits controlled by a combination of allelic variants and environmental factors. GeneNetwork with its central module WebQTL facilitates the exploitation of permanent genetic reference populations that are accompanied by genotypic, phenotypic and mRNA abundance datasets. Algorithms for both quantitative trait locus (QTL) mapping and genetic correlation analysis, supported by highly efficient graphical displays facilitate the identification of QTL controlling mRNA transcript abundance (expression-QTL or eQTL) and higher-order phenotypes. Consequently, GeneNetwork is an unique on-line environment for 'trait analysis' at the systems biology level [18,19].

One of our long term goals is to construct integrated regulatory and structural gene association networks that explain relationships between component gene expression measures and traditional phenotypic traits. We have started this by constructing a trait association network to establish connections and to provide a framework for the identification and mapping of key regulatory genes. Here we describe these barley data sets and demonstrate how GeneNetwork's integrated analytical environment can be exploited to infer map positions of the barley genes and to construct barley trait association networks.

## Methods

### Database schema

Construction of the database underlying GeneNetwork for mouse data sets has been described previously [18,19]. Database schema and description is available from [20].

### The current barley data set in GeneNetwork

A population of 150 doubled haploid lines (DHLs) derived from a cross between cultivars (cvs.) Steptoe and Morex (St/Mx) was used to generate the mRNA transcript abundance, trait and genotypic data sets. These parents were selected because of their diversity for agronomic traits [21]. Steptoe is a high yielding, broadly adapted six-rowed feed-type barley from the Western United States (US), whereas Morex is a six-rowed malting cultivar from the Midwestern US.

#### Phenotypic traits

We have compiled and integrated into GeneNetwork data corresponding to 23 phenotypic traits, fifteen of them not published previously (Table 1). For the phenotypic data obtained from plants grown in the east of Scotland from 2002–2005, we maintained individual field trial data scores as separate entries. Similarly, for the published set of 8 traits [22], measured in 9–16 locations across the US and Canada, we kept the data from each location as a separate entry. For the rest of the traits that have replicate measurements, arithmetic mean, standard deviation and the number of replications were entered into GeneNetwork, thus enabling the use of variance for weighted regression analyses. The total count of individual higher-order phenotypic barley trait entries in GeneNetwork is 211.

**Table 1:** Condensed list of barley traits that have been measured using the Steptoe × Morex DHL population and are available for analysis through GeneNetwork.

**DEVELOPMENTAL TRAITS**			
***Trait ID***	***Method/equipment***	***Location***	***Reference***

Germination	Frequency of the germinating grains	10	cp
Emergence of the second leaf (ESL)	Single-leaf frequency (ESL-f) and length ratio of the second and the first leaf (ESL-r)	3	cp
Heading date	Time interval to heading or anthesis	1–16, 17–20	cp, [22]
Height	Distance from ground to collar at maturity	1–16, 17–20	cp, [22]
Lodging	Stems < 45 degree angle to ground (1–9)	1–16, 17–20	cp, [22]
Maturity	Maturity of the plot (1–9)	17–20	cp
Normalized difference vegetation index (NDVI)	GREENSEEKER	17–20	cp
Head length	Distance from peduncle to the awn tip	17–20	cp
Post harvest sprouting	Frequency of the germinating grains	10	cp
Head loss	Frequency of the tillers with no heads	3	cp
Grain loss	Frequency of the heads with no spikelets.	3	cp
Thousand grain weight (TGW).	TGW = 1000 × weight/seed number.	17–20, 25	cp
Grain morphometrics.	MARVIN and ImageJ	17–20, 25	cp
			
**QUALITY TRAITS**			
			
Endosperm cell wall modification.	Calcuflor staining, ImageJ analysis	20	cp
Nitrogen content or grain protein	Micromalting	1–20	cp, [22]
Malt extract	Micromalting	1–22	cp,[22]
Fementability	Micromalting	1–3	cp
Hot water extract	Micromalting	1–3	cp
Milling energy (J)	COMPARAMILL	1–3	cp
Predicted spirit yield	Micromalting	1–3	cp
Grain moisture content	Moisture content of sample %	1–3	cp
Diastatic power	Micromalting	7–22	[22]
Alpha amylase	Micromalting	7–22	[22]
			
**INTERACTION WITH PATHOGENS**			
			
Leaf rust (Puccinia hordeii)	Relative Latency Period	24	[60]
Net bloch (Pyrenophora teres)	Frequency of the infection types	6	[61]
Scald (Rhyncosporium secalis)	Disease severity	25	cp
Spot bloch (Cochliobolus sativus)	Frequency of the infection types	6	[62]
Stem rust (Puccinia graminis)	Frequency of the infection types	6	[13]

Detailed descriptions of the traits are here:

*Environments/years/source:*

1 – SCRI, 2002; 2 – SCRI, 2003; 3 – SCRI, 2004; 4 – SCRI, 2005; 5 – SCRI; 2006; 6 – Minnesota; 7 – Crookston, Minnesota; 8 – Ithaca, New York; 9 – Guelph, Ontario; 10 – Pullman, Washington; 11 – Brandon, Manitoba; 12 – Outlook, Saskatchewan; 13 – Goodale, Saskatchewan; 14 – Saskatoon, Saskatchewan; 15 – Tetonia, Idaho; 16 – Bozeman, Montana (irrigated); 17 – Bozeman, Montana (dryland); 18 – Aberdeen, Idaho; 19 -Klamath Falls, Oregon; 20 – Pullman, Washington; 21 – Bozeman, Montana (irrigated); 22 – Bozeman, Montana (dryland); 23 – Japan (Kazuhiro Sato, seeds for morphometrics); 24 – Wageningen; 25 – Giessen. cp – current paper.

#### mRNA transcript abundance data

There are two barley transcript abundance data sets available for analysis in GeneNetwork – a set of 139 lines of embryo-derived tissues, and a set of 30 seedling leaf samples. The raw data (Affymetrix' CEL files) and all 22,840 Barley1 GeneChip signal values calculated using either RMA or MAS5.0 algorithms [23] using Genespring 7.3 (Agilent Technologies, Inc.) were incorporated into GeneNetwork (Table 2). Originally, profiling of embryo-derived tissues was done using 150 lines and seedling leaf using 35 lines. However, 11 lines had ambiguous genotypes, suggesting mishandling at some stage, and therefore were removed from the dataset [17].

**Table 2:** Barley expression data sets available for analysis in GeneNetwork.

**Types of the expression data sets**	**Data processing description**
***Barley1 Embryo*** ***gcRMA SCRI*** ***(Dec 06)*** ***Barley1 Leaf*** ***gcRMA SCRI*** ***(Dec 06)***	The Affymetrix' CEL files that were generated using MAS 5.0 Suite (Affymetrix, Santa Clara, CA) were imported into the GeneSpring GX 7.3 (Agilent Technologies, Palo Alto, CA) and processed using the RMA algorithm.
	
***Barley1 Embryo*** ***MAS 5.0 SCRI*** ***(Dec 06)*** ***Barley1 Leaf*** ***MAS 5.0 SCRI*** ***(Dec 06)***	The MAS 5.0 values were calculated from the DAT files using Affymetrix' MAS 5.0 Suite (Affymetrix, Santa Clara, CA).
	
***Barley1 Embryo0*** ***gcRMA SCRI*** ***(Apr 06)*** ***Barley1 Leaf*** ***gcRMAn SCRI*** ***(Dec 06)***	The Affymetrix' CEL files were imported into the GeneSpring GX 7.3 (Agilent Technologies, Palo Alto, CA) software and processed using the RMA algorithm. Per-chip and per-gene normalization was done following the standard GeneSpring procedure which includes setting the values below 0.01 to 0.01 and then dividing each measurement by the 50th percentile of all measurements in that sample. Additionally each gene was divided by the median of its measurements in all samples.

#### Genotypes

The linkage map presented here was generated as part of two barley association mapping projects in the United Kingdom (UK) [24] and US [25] (also [26,27]). To create the genotype file, we used data from a pilot barley Illumina Oligo Pool Assay (POPA1) that employs GoldenGate BeadArray technology (Illumina, SanDiego CA) and tested 1,536 barley SNP markers in each of the 150 St/Mx DHLs. 471 high quality polymorphic SNPs were integrated into the existing St/Mx RFLP map [21] using Map Manager QTX (ver. 0.27) software [28]. A final map was generated by removing co-segregating markers (leaving a single marker per locus) and manually checking and correcting the relatively rare single marker double recombination events visible in graphical genotypes of the individuals in the population.

## Discussion

### Using GeneNetwork for barley

**Figure 1:**
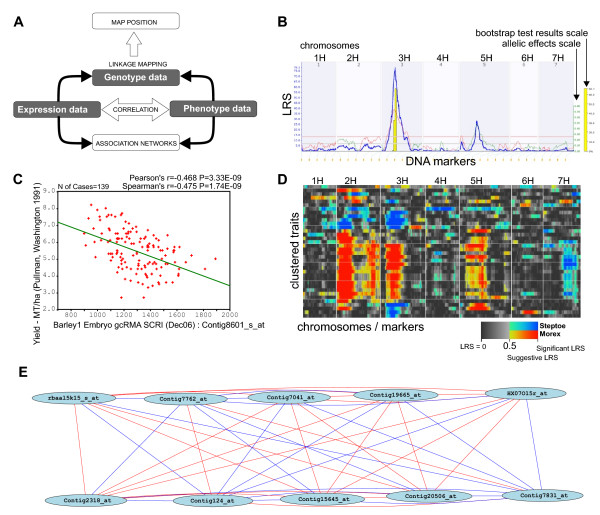
**A – Generalized schematic representation of the functions and their relationships in GeneNetwork related to three types of data; gene expression, phenotype and genotype**. B-E examples of typical graphical outputs generated by the GeneNetwork. B – Profile of a QTL scan using the interval mapping function. The blue line graph – Likelihood Ratio Statistic (LRS) profile, green and red line graphs – allelic effects (in our case green = Morex, red = Steptoe), yellow bars – confidence intervals determined using 1000 bootstrap tests, red and grey horizontal lines – upper and lower significance LRS thresholds determined by 1000 permutation tests; C – Any pairwise correlation can be visualized as a scatter plot allowing the correlation structure to be determined. In this case, mRNA abundance values (reported by the GeneChip probe set Contig8601_s_at) were plotted against grain yield values from one of the trials. 'N of cases' – number of segregating lines. Pearson's and Spearman's correlation coefficients and associated p-values (P) are shown on the top right corner. Linear regression line is shown in green.; D – Selected correlates can also be visualized as a QTL Cluster map, which is a genetically ordered heat-map representation of the QTLs from multiple traits that were calculated using single marker linkage analysis. Significant QTLs are shown in a different colour from loci that have no association, and allelic effects are shown in contrasting colours (red and blue in key). E – Association network of 10 correlated genes. As a 'seed', mRNA abundance of the HLH DNA-binding protein gene (Contig20506_at), was used. Pearson's correlation coefficient threshold in this case was |0.8|. Line colours show correlation strength (more intense – higher correlation) and whether it is positive (orange – red) or negative (green – blue).

The framework for analysis using GeneNetwork for barley is shown in Figure 1A. Associations between transcript abundance, phenotypic traits and genotype can be established either using correlation or genetic linkage mapping functions [29,30]. The main page of GeneNetwork at  provides access to subsets of data through pull-down menus that allow specific data sets to be queried. The datasets can be further restricted using a single text box for specific database entries to query probe set or trait ID, or annotations associated with the database entries. Once the resulting record set of the query is returned, it can be further restricted by selecting relevant records based on attached annotations before forwarding it for further analysis.

To map genetic loci associated with mRNA abundance or trait phenotypes, any one of the three QTL mapping functions currently employed by GeneNetwork's WebQTL module can be used. These are 1. interval mapping, 2. single-marker regression, or 3. composite mapping [29,30]. A thousand permutations are used to calculate upper and lower Likelihood Ratio Statistic (LRS) thresholds for each trait [31], and 1000 bootstrap tests [32,33] can be employed to determine the confidence intervals (Figure 1B).

The correlation analysis module performs either Pearson product-moment correlation or Spearman rank correlation. Different trait and transcript abundance values (either as integrated or individual probe signals) as well as genotypes can be used to correlate against other data sets of choice. Results of the correlation analyses can be displayed as a table showing correlation coefficients and p-values. The covariates can then be visualized pair-wise as scatter plots (Figure 1C), mapped using the QTL Cluster function (Figure 1D) or combined into association networks [34,35] (Figure 1E).

### Predicting gene position

One of the basic, but arguably most relevant applications of GeneNetwork for barley is to predict the map location of a gene. Until its genome is sequenced or all known barley genes are mapped as genetic markers (e.g. SNPs), the ability to infer a gene's chromosomal position (with a given degree of certainty) by mapping the genetic interval that controls the abundance of its mRNA (as an eQTL) provides valuable information about location of the gene itself. This is easily achieved in the GeneNetwork using its integrated QTL mapping functions.

**Figure 2:**
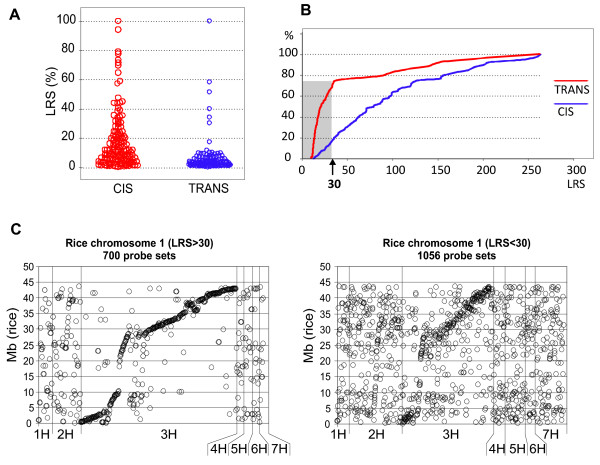
**Prediction of barley gene position based on linkage analysis of mRNA abundance**. A – Scattergram of the LRS value distributions of 324 eQTLs with genetic positions of the underlying genes determined using SNP- or RFLP-based linkage mapping. B – Cumulative (%) distribution of the LRS values for *cis*- (blue line graph) and *trans*- (red line graph) eQTLs. C – Scatterplots showing the distribution of high (> 30) and low (< 30) LRS class eQTLs across the barley genetic map (x-axes) relative to the position of their putative rice orthologs. Each diagram shows only the comparison to rice chromosome 1 which exhibits considerable conservation of synteny with barley 3H (y-axes). On the x-axis the eQTL positions of barley orthologs of genes on rice chromosome 1 are ordered according to their location on the barley genetic map (tip of barley 1HS to bottom of 7HL), but barley map distances are not taken into account. As expected, barley 3H exhibits strong synteny with rice 1. This is particularly obvious when considering the eQTLs with LRS > 30, suggesting that this class of eQTLs is generally *cis-*acting. The eQTLs with LRS < 30 show a less obvious (but still apparent) association between rice 1 and barley 3H. In these comparisons all genes reported by 22,840 Barley1 GeneChip probe sets were analysed.

When an eQTL is described by a single peak that coincides with the gene's location, then variation in *cis*-regulatory elements that control the expression of the associated gene is the most likely explanation. Alternatively, if the structural gene is located distantly from its eQTL peak, then the eQTL may represent the location of a regulatory factor, which affects the abundance of the monitored mRNA (i.e. a *trans*-regulator). One possible approach to inferring *cis*- vs. *trans*- regulation, and hence the gene's approximate position is based on the experimentally tested observation that strong eQTL (LRS > 30–40) are typically *cis*- regulated [3]. The scattergram in Figure 2A partitions 345 previously mapped genes into *cis*- and *trans*- eQTLs according to co-location of their structural genes and eQTLs (see also additional file 1). It shows that most eQTLs with an LRS>30 (~20% on the scattergram) are likely to be regulated as *cis*- (Figure 2B). It also shows that the prediction of *trans-*regulated genes can not be made using this approach because many *cis-*regulated genes are in the same LRS value range as *trans-*regulated genes.

Support for this simple designation of a gene's map location comes from an analysis of conserved synteny between the rice genome sequence and the barley gene map. The rationale is that an eQTL will more likely reflect the true position of its underlying gene if its rice ortholog is located in the conserved syntenic position. We sub-divided all the probe sets that reported significant eQTLs into the high (LRS > 30) and low (LRS < 30) LRS groups and plotted their barley eQTL peak positions against the physical positions of their putative rice orthologs (Additional file 2). For 9 out of 12 rice chromosomes, clear blocks of conserved synteny were revealed with eQTLs with high LRS values, whereas many low LRS value eQTLs were homogenously distributed across the rice genome (for example rice chromosome 1 in Figure 2B). Conservation of synteny provides additional support for the principle of mapping a barley gene based on QTL mapping of mRNA abundance values.

### Constructing trait association networks

An association network for a given set of traits is a graphical display of all pair-wise correlations that are above an arbitrarily assigned correlation threshold value [36]. GeneNetwork has a function that constructs such association networks using either phenotype or transcript abundance, or indeed both simultaneously. It provides a visualization of the relative positions and numbers of possible interacting partners, how they interact (positive or negative correlation) and in some situations, based on prior knowledge, it may suggest the directionality of the interaction.

**Figure 3:**
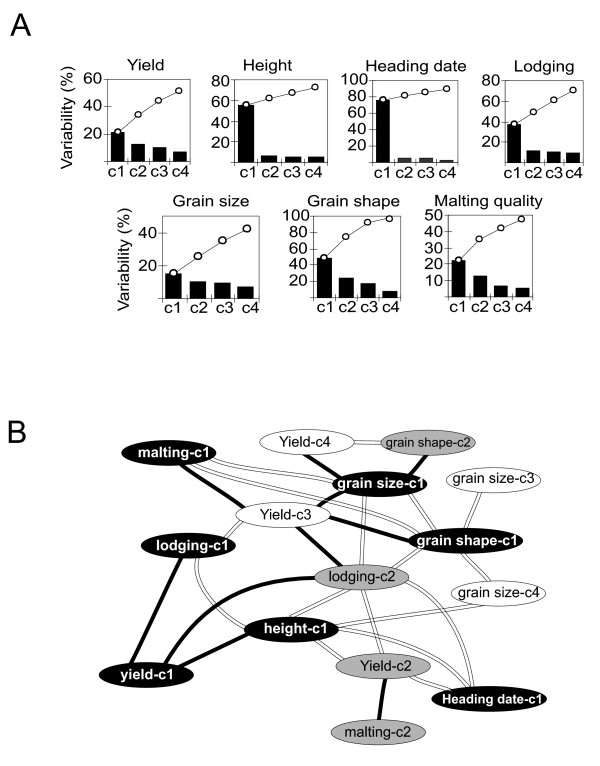
**Results of principal component analysis (A) and association network (B) show the relationships between the major barley phenotypic traits integrated into the GeneNetwork**. The network was built using scores of the first four principal components (c1–c4) calculated by combining data from a single trait measured in different locations and years, or related (component) traits underlying a higher order trait (e.g. malt quality data). Concerning the latter, principal component scores for malting quality traits were calculated from combined alpha amylase, diastatic power, grain protein and malt extract trait values. Principal component node colouring; c1-black background, c2-grey, c3 and c4 – white). Double-lined links – positive correlations; Bold, thick links – negative. For clarity, the network was re-drawn using GeneNetwork's output.

An association network using principal component scores calculated using a selected set of malting quality and yield-related trait data as variables provides an overview of the key barley traits that segregate in the St/Mx population (Figure 3, Additional File 3). The cumulative variation explained by the first four principle components ranged from around 90% for heading date to 40% for grain size (Figure 3A), suggesting a strong genetic component for the former, and a more complex situation for the latter. The derived association network (Figure 3B) revealed some known and obvious relationships. For example, the main yield component 'yield-c1' (c1 = principle component 1) is negatively correlated with 'plant height-c1' and 'lodging-c1' and 'lodging-c2'. In contrast, there is a positive correlation between 'lodging-c1' and -c2 with 'height-c1'. This is entirely consistent with taller plants lodging more which results in grain loss during harvest. The St/Mx population was originally designed to dissect two contrasting barley traits, yield and malting quality [21]. The trait association network in Figure 3B shows links only between the minor components of these traits (malting-c1 to yield-c3 and malting-c2 to yield-c2) suggesting complex underlying genetics.

Since association networks are based on correlation, they differentiate neither causal from reactive traits, nor genetic from environmental factors. Genetic linkage mapping, of course, can provide this distinction if a mapping population with sufficiently high resolution is used and sufficient replication is incorporated in the experimental design. Furthermore, in the case of transcript abundance traits, the integration of data from 'classical' or 'treatment-response' type profiling experiments as well as fine scale haplotype map information may clarify the difference between causal and reactive traits [5]. However we note that there is an extra layer of complexity when dealing with an unsequenced genome. Without knowing the regulatory genes underlying key phenotypic traits, and without having precise map positions for the majority of the genes, it is critical that any mRNA abundance based association network analysis is conducted with caution and stringent validation strategies deployed to support any putative links.

### Future developments

The GeneNetwork is an acknowledged and widely used integrated platform designed primarily for analysis of data from mouse genetical genomics experiments [18,19,36]. In the future we intend to integrate mRNA profiling, phenotypic and genotypic data from alternative populations that have a different genetic architecture along with molecular profiling data, such as proteins or metabolites, together with access to gene and pathway models and annotations from model plant genomes.

Incorporating algorithms and data handling functions for mapping dynamic traits, also known as functional mapping [38,39] is also a priority. The approach has been applied to diverse range of species, including humans, animals and plants, to uncover novel information [38,40-46]. However, to our knowledge, there are no available barley data sets that are suitable for dynamic trait mapping. Preliminary experiments on grain development [47] and interactions with pathogens [48-51] provide examples and methodologies for obtaining trait values that could be easily applied to an expanded sample population, however, this hasn't been done yet. Functional mapping of data relating to classical traits such as height, flowering time and malting quality could also reveal novel QTL or relationships between existing QTL. However, this knowledge will only improve our understanding of the causal biological process if the genes underlying the QTL are cloned.

The collection of precise phenotypic data across a population and over time would reveal more significant QTL and provide a better link to 'surrogates' such as mRNA abundance, especially if the latter was derived from specific and relevant cell types. As an example, endosperm modification is a key barley quality trait central to both malting and distilling. We mapped endosperm modification as the area ratio of endosperm stained with calcuflor to the unstained area. Calcuflor stains polymeric 1,3–1,4 -beta glucans which are important barley cell wall constituents and their amount decreases when the cell walls are broken down by cellulytic enzymes. The collection of calcuflor staining data on a population of plants over time is an eminently feasible experiment and would allow endosperm modification to be considered as a dynamic trait with the obvious potential of revealing novel QTL controlling biochemical processes activated during germination.

The object models underlying GeneNetwork have been designed for handling data linked to a well established, stable sequencing data that for the mouse have been available for years. For barley and other less thoroughly researched species this is still in a distant future. This is viewed as a major hindrance for high level genetical genomics analysis by many researchers. However, we were able to integrate barley data in the software designed for mouse without any changes to the software itself and just minor adjustments to the existing barley data. This suggests that software that is designed according to the nature of the biological object can be easily adopted to work with objects of the same kind but lacking some essential property values. Therefore the lack of sequence shouldn't be an obstacle for genetical genomics analysis. By integrating datasets from an unsequenced crop plant (barley) in a database that has been designed for an animal model species (mouse) with well established genome sequence, we prove the importance of the concept and practice of modular development and interoperability of software engineering for biological data sets.

Linking barley data in the GeneNetwork to other relevant genomic resources, such as the Barley SNP Database (SNPDb) [52], Harvest [53], BarleyBase (within PLEXdb) [54], GrainGenes [55] and Gramene [56] will significantly enhance the interpretation of the molecular basis of higher order phenotypes in barley. The success of this implementation largely depends on the development of flexible and streamlined data processing and submission procedures that can handle heterogeneous data types and provide efficient cross-referencing. XML-based technologies seem well suited to handle this [57].

## Conclusion

By integrating barley genotypic, phenotypic and mRNA abundance data sets directly within GeneNetwork's analytical environment we provide simple web access to the data for the research community. In this environment, a combination of correlation analysis and linkage mapping provides the potential to identify and substantiate gene targets for saturation mapping and positional cloning. By integrating datasets from an unsequenced crop plant (barley) in a database that has been designed for an animal model species (mouse) with well established genome sequence, we prove the importance of the concept and practice of modular development and interoperability of software engineering for biological data sets.

## Availability and requirements

GeneNetwork usage conditions and limitations are available from here [58]. Online tutorial accompanying this manuscript can be either viewed or downloaded from the [59].

## Authors' contributions

AD concept, phenotyping, data entry, drafting the manuscript; ID concept, data processing, data entry, image analysis, writing; AGC data processing, data entry; HL data processing, data entry; ZS data processing, data entry; WTT phenotyping; NB SNP mapping, phenotyping; BJS phenotyping; SEU phenotyping; AK RFLP mapping; RPW RNA labelling, GeneChip processing; TJC SNP Illumina genotyping; EP QTL mapping; ZL SFP detection; CW phenotyping; GFS phenotyping; DFM rice-barley comparison; MJK project PI; concept; RWW concept, data processing, data entry; RW project PI, concept, writing.

## Supplementary Material

Additional file 1 Table S1. Inference of mRNA abundance regulation by *cis*-elements or *trans*-factors. This is a tab delimited table, the first row contains column headings. 'Cosegregating marker' – DNA marker ID that co-segregates with the gene underlying the 'Probe set'. 'Probe Set' – Affymetrix' Barley1 GeneChip probe set ID. 'DNA marker chromosome' and 'DNA marker position' – 'Cosegregating marker' locus parameters. 'mRNA abundance QTL chromosome', 'mRNA abundance QTL position' and 'LRS' – mRNA abundance QTL parameters of the gene underlying 'Probe set'. LRS – Likelihood Ratio Statistic. 'cis/trans' – c – *cis*, t – *trans*, inference on *cis*- or *trans *regulation of the gene underlying 'Probe set'. Click here for file

Additional file 2 Table S2. The Barley1 GeneChip probe sets that report significant mRNA abundance QTL and have rice homologs for the underlying genes. This is a tab delimited table, the first row represents column headings. 'ProbeSet' – Affymetrix' Barley1 GeneChip probe set IDs. 'LRS' – LRS (Likelihood Ratio Statistic) of the mRNA abundance QTL reported by the 'ProbeSet'. 'LRS_range' – subdivision of the LRS into 'low' and 'high' groups. 'Locus' and 'barley chromosome/marker' – location parameters of the mRNA abundance QTL. 'Rice Chr' and 'Rice 5" – location parameters of the rice homologs. Click here for file

Additional file 3 Table S3. Principal Components scores of the key traits that segregate in the SM population. This is a tab delimited file, first row represents column headings. F1–F4 represent factors of individual traits. Click here for file
